# Altered brain entropy and functional connectivity patterns in peritoneal dialysis patients

**DOI:** 10.3389/fnins.2026.1877959

**Published:** 2026-07-09

**Authors:** Dashan Li, Zilian Chen, Luyao Yu, Yongjie Yin, Jie Fang, Xiangming Qi, Haibao Wang, Yonggui Wu

**Affiliations:** 1 Department of Nephropathy, The First Affiliated Hospital of Anhui Medical University, Hefei, Anhui, China; 2 Department of Radiology, The First Affiliated Hospital of Anhui Medical University, Hefei, Anhui, China

**Keywords:** cognitive impairment, functional connectivity, functional magnetic resonance imaging, peritoneal dialysis, sample entropy

## Abstract

**Objective:**

To explore abnormal changes in brain entropy (BEN) and resting-state functional connectivity (RSFC) in peritoneal dialysis (PD) patients and their associations with cognitive impairment (CI).

**Methods:**

Fifty-three PD patients and 49 age-, gender-, and education-matched healthy controls (HCs) were enrolled. Resting-state functional magnetic resonance imaging (rs-fMRI) was performed to calculate BEN and RSFC. Neuropsychological assessments and clinical indicator collection were conducted. PD patients were divided into mild cognitive impairment (MCI) and non-cognitive impairment (NCI) groups using Montreal Cognitive Assessment (MoCA) scores. Correlation analyses were performed between BEN/RSFC values and neuropsychological/clinical indicators.

**Results:**

PD patients exhibited significantly poorer performance in multiple cognitive scales than HCs (all *p* < 0.001). Compared with HCs, PD patients had decreased BEN in the right middle occipital gyrus and left caudate nucleus, and increased BEN in the left middle temporal gyrus and right fusiform gyrus. Reduced RSFC was found between the right middle occipital gyrus and the right fusiform gyrus, right middle frontal gyrus, and right precuneus in PD patients. BEN and RSFC values were correlated with emotional scale scores, cognitive scale subscores, and clinical indicators (e.g., glycosylated hemoglobin, transferrin saturation).

**Conclusion:**

Patients with end-stage kidney disease undergoing peritoneal dialysis present abnormal brain entropy and functional connectivity patterns. These alterations are associated with systemic metabolic disorders, long-term dialysis treatment, and cognitive/emotional impairment.

## 1 Introduction

Peritoneal dialysis (PD) is a major replacement therapy for patients with end-stage kidney disease (ESKD) secondary to chronic kidney disease (CKD). Cognitive impairment (CI) is a common complication among PD patients and an independent mortality risk factor for ESKD individuals (Angermann et al., 2018). Previous studies reported that the prevalence of CI in PD patients ranges from 27 to 70%, and over half of these cases are moderate to severe (Angermann et al., 2018; Murray et al., 2006). Cognitive impairment not only affects their medical compliance and quality of life but also may lead to the occurrence of PD-related peritonitis, thereby increasing the hospitalization rate and mortality of patients. Therefore, early identification and intervention for CI in PD patients are clinically important.

The human brain is recognized as a complex nonlinear system with a hierarchical structure (Jia and Gu, 2019). Brain entropy (BEN), calculated based on blood-oxygen-level dependent (BOLD) signals, is a nonlinear metric that reflects the complexity of brain activity and information processing (Sokunbi et al., 2014; Yentes et al., 2013). In resting-state functional magnetic resonance imaging (rs-fMRI), BEN can quantify spontaneous brain fluctuations and has been regarded as a potential neuroimaging marker for neurological and psychiatric disorders (Saxe et al., 2018). This method has been widely applied in multiple brain disease studies. For instance, the calculation of Sample Entropy (SampEn) of the Center of Pressure (CoP) displacement before and after the transition between sitting and standing postures in patients with frailty syndrome can reflect the nonlinear characteristics of their postural control (Vassimon-Barroso et al., 2017). The reduced entropy in the occipital, frontal and temporal lobes was found in Alzheimer’s disease (Wang et al., 2017) and correlated with cognitive decline; altered sample entropy also revealed abnormal brain activity in patients with cerebral small vessel disease. (Zhang et al., 2024). Currently, mainstream rs-fMRI indicators for brain function evaluation mainly include amplitude of low-frequency fluctuation (ALFF) and regional homogeneity (ReHo), which are linear static measurements. Differently, BEN reflects the nonlinear dynamic characteristics of the brain. However, no study has explored the correlation between BEN changes and cognitive function in PD patients.

Given the above research gaps, we performed rs-fMRI examinations to characterize abnormal brain entropy and resting-state functional connectivity in peritoneal dialysis patients. The core objectives of this study were to clarify the overall differences in brain functional activity between peritoneal dialysis patients and healthy individuals, and to analyze the correlations of brain entropy and functional connectivity with cognitive performance, emotional symptoms and routine clinical parameters. Our results may offer a novel research perspective for the study of cognitive impairment associated with peritoneal dialysis.

## 2 Methods

### 2.1 Study subjects

ESKD patients who were hospitalized in the Department of Nephrology, the First Affiliated Hospital of Anhui Medical University, and received regular PD from June 2024 to March 2025, meeting the inclusion and exclusion criteria, were selected. Sample size was calculated using the formula for cross-sectional research:

$$n=\frac{Ζ\alpha^{2}\times P\times (1-P)}{d2}$$

With a prevalence of 60%, a 15% margin of error and *α* = 0.05, the minimum sample size was 41 cases. Including a 10% dropout rate, the required sample size was adjusted to 46 cases. Inclusion criteria: (1) Aged 20–70 years, receiving continuous ambulatory peritoneal dialysis with a stable dialysis duration of more than 3 months; (2) Right-handed with no communication barriers. Exclusion criteria: (1) A history of craniocerebral injury and other brain diseases, such as cerebral hemorrhage, traumatic brain injury, and intracranial space-occupying lesions; (2) A documented clinical diagnosis of major psychiatric disorders (e.g., major depressive disorder, generalized anxiety disorder, schizophrenia) according to medical records, regardless of current treatment status. It should be noted that subclinical anxiety or depressive symptoms measured by the Self-Rating Anxiety Scale (SAS), Self-Rating Depression Scale (SDS), and 9-item Patient Health Questionnaire (PHQ-9) did not lead to exclusion; instead, these scores were collected for subsequent correlation analyses. (3) A history of substance dependence or abuse, including alcohol, drugs (including anticholinergics, benzodiazepines, antidepressants, opioid analgesics, and first-generation antihistamines), etc.; (4) A history of kidney transplantation, or presence of acute infection, heart failure, or severe liver function damage during hospitalization; (5) Inability to cooperate with neuropsychological assessments and cranial magnetic resonance examinations; (6) Presence of obvious intracranial organic lesions found in routine T2 magnetic resonance examination, such as cerebral softening focus, cerebral infarction, white matter lesions, etc. A total of 53 PD patients were enrolled in this study, including 19 males and 34 females, with an average age of (50.83 ± 7.90) years. Healthy adults matched with the above PD patients in terms of age, gender, and educational level were selected. A total of 49 healthy controls were included, including 15 males and 34 females, with an average age of (49.37 ± 8.12) years.

This study is a cross-sectional study, which has been approved by the Ethics Committee of the First Affiliated Hospital of Anhui Medical University. All subjects were fully informed in writing or verbally before enrollment and provided their informed consent.

### 2.2 General information and neuropsychological assessments

General information: Age, gender, dialysis duration (months), years of education, and other comorbid diseases such as hypertension, diabetes, and coronary heart disease were collected for both the PD group and the normal control group. We collected the primary etiologies of ESKD and long-term regular medications of PD patients, including antihypertensives, hypoglycemic drugs, iron preparations, and erythropoietin.

Laboratory data: For patients in the PD group, indicators such as hemoglobin, albumin, blood urea nitrogen (BUN), uric acid, serum creatinine, calcium-phosphorus product, glycosylated hemoglobin, transferrin, transferrin saturation, unsaturated iron-binding capacity, total cholesterol, high-density lipoprotein cholesterol (HDL-C), low-density lipoprotein cholesterol (LDL-C), triglycerides, and intact parathyroid hormone (iPTH), etc. were collected. Additionally, we collected indicators reflecting residual renal function and dialysis adequacy for all PD patients, including 24-h residual urine volume, total Kt/V (urea clearance index) and weekly creatinine clearance (CCr). No blood biochemical tests were performed on the control subjects.

Neuropsychological tests were conducted on eligible PD patients and normal controls by professionally trained assessors with rich experience in a quiet environment free from external interference, strictly following the scale operation manuals. Various cognitive functions of the subjects were assessed, including: (1) Global cognitive function: Montreal Cognitive Assessment (MoCA), Mini-Mental State Examination (MMSE); (2) Executive function: Stroop test, Digit Span Test (DST), Trail Making Test (TMT); (3) Attention function: Symbol Digit Modalities Test (SDMT); (4) Language function: Verbal Fluency Test (VFT); (5) Emotional assessment: SAS, SDS, PHQ-9. PD patients were divided into MCI group and non-cognitive impairment (NCI) group based on MoCA scores. The MoCA assesses cognition, including 7 sub-cognitive domains: visuospatial and executive function, naming, delayed recall, attention, language, abstract thinking, and orientation, with a total score of 30 (Lu et al., 2011). The score is adjusted according to educational level: ifyears of education is ≤ 12 years, 1 point is added to the total score, with the total score not exceeding 30. The cut-off value is 26 points; a score < 26 is considered to indicate MCI, and a lower score indicates poorer cognitive function (Marcos et al., 2016).

### 2.3 MRI data acquisition

MRI data of all subjects were acquired using a 3.0 T MRI system (Siemens Healthcare, Erlangen, Germany) equipped with a 64-channel head matrix coil. Before scanning, it was ensured that patients had no metal objects on them, and earplugs were provided to reduce the impact of noise on patients. Subjects were instructed to remain as still as possible during scanning, close their eyes without falling asleep, and try not to think about anything. After the patient lay on the scanning bed, foam pads were used to assist in fixing the head to reduce the impact of excessive head movement on image quality. The scanning sequences mainly included: 3D-T1 whole-brain structural sequence, rs-fMRI sequence, and T2-FLAIR sequence. The specific scanning parameters are as follows: T2-FLAIR: echo time 98 ms, repetition time 8,000 ms, field of view 220 mm × 200 mm, flip angle 150°, matrix 320 × 224, slice thickness 5 mm, slice gap 1 mm, number of slices 24, scanning time 98 s; 3D-T1 (structural image): echo time 2.9 ms, repetition time 2,300 ms, field of view 256 mm × 240 mm, flip angle 9°, matrix 256 × 256, slice thickness 1 mm, slice gap 0.5 mm, number of slices 208, scanning time 5.1 min; rs-fMRI (resting-state functional image): echo time 30 ms, repetition time 3,000 ms, field of view 220 mm × 220 mm, flip angle 90°, matrix 64 × 64, slice thickness 3.4 mm, slice gap 0 mm, number of slices 48, total 197 time points, scanning time 10 min.

### 2.4 Magnetic resonance data preprocessing

Data preprocessing was performed using the Resting-State Functional Magnetic Resonance Imaging Toolkit (DPARSF, http://rfmri.org/dpabi) and SPM12 (http://www.fil.ion.ucl.ac.uk/spm) on the MATLAB platform (Yan et al., 2016). To eliminate the interference caused by machine and subject adaptation at the beginning of the test, the first 10 time points of the images were removed. Temporal slice correction was performed on the images to reduce time errors introduced by inter-slice scanning. Images were realigned to the middle slice for head motion correction; subjects were excluded if head motion exceeded 3 mm or 3°. Subsequently, the remaining images were processed through the following steps: functional images were registered to the Montreal Neurological Institute (MNI) template space (resampled to 3 × 3 × 3 mm voxels), confounding regression variables (Friston-24 head motion parameters, white matter hyperintensities, and cerebrospinal fluid signals) were removed, and temporal filtering (0.01–0.1 Hz) was performed.

### 2.5 Gray matter volume analysis

Structural MRI data were preprocessed using the voxel-based morphometry (VBM) module in the Computational Anatomy Toolbox (CAT12, version r2577). Three-dimensional T1-weighted images underwent spatial normalization and tissue segmentation to generate total intracranial volume (TIV) and gray matter volume (GMV).

### 2.6 Brain entropy calculation

The sample entropy value of each voxel was calculated using the Brain Entropy Mapping Toolbox (BENtbx1) combined with an open-source brain entropy plotting toolbox (Wang et al., 2014). In this study, m represents the pattern length, r represents the tolerance value, and N represents the length of the time series. Based on previous studies, m was set to 3 and r to 0.6. Brain entropy values were calculated voxel-wise across the entire brain, and Gaussian smoothing with a 6 mm kernel was applied. Statistical analysis: Using gender, age, and educational level as covariates, two-sample t-tests were performed on brain entropy maps of normal subjects and patient groups using SPM12 to obtain brain regions with statistically significant differences between the two groups (voxel-level *p* < 0.001, FWE correction), and Fisher-z transformation was applied to improve normality, which were regarded as regions of interest. Then, sample entropy values within the regions of interest (ROI) were extracted and correlated with patients’ neuropsychological scales and clinical indicators, respectively.

### 2.7 Calculation of functional connectivity in regions of interest

The above-obtained ROIs were made into masks. Whole-brain voxel-wise functional connectivity (FC) based on ROIs was performed in REST’s Fun connectivity module. Then, the Pearson correlation coefficient between the time series extracted from each ROI and the time series of each voxel in the entire brain for each subject was calculated to obtain correlation maps, and Fisher-z transformation was applied to improve normality for subsequent statistical analysis. Statistical analysis: First, two-sample T-tests were performed on zFC maps of ROI and whole brain between normal controls and PD patients using SPM12 (voxel-level *p* < 0.001, FWE correction) to obtain brain regions with differences in each ROI between the two groups of subjects. Meanwhile, correlation analyses were conducted between FC values of each ROI and differential brain regions with patients’ neuropsychological scales and clinical indicators, with a threshold of *p* < 0.05.

### 2.8 Statistical methods

SPSS statistical software (version 25.0) was used for statistical analysis in this study. Continuous variables between groups were expressed as mean ± standard deviation; independent sample t-tests were used for inter-group comparisons if they conformed to a normal distribution, otherwise, rank-sum tests were used. Categorical variables were expressed as cases (percentiles), and chi-square tests were used for inter-group comparisons. Pearson or Spearman correlation tests were used for correlation analyses. Additionally, partial correlation analyses with age, educational level and gray matter volume as covariates were performed to control potential confounding influences. A two-tailed *p* < 0.05 was considered statistically significant.

## 3 Results

### 3.1 General clinical data and neuropsychological assessment

A total of 53 patients receiving regular PD were enrolled in this study as the PD group, and 49 age-, gender-, and education-matched healthy adults were recruited during the same period as the HC group. There were no statistically significant differences in general demographic data such as age, gender, and years of education between the two groups (*p* > 0.05). In terms of cognitive function, the results of the MoCA, MMSE, SDMT, Stroop Test, DST, VFT, SAS, SDS, and PHQ-9 in the PD group were significantly worse than those in the healthy control group (all *p* < 0.001) (Table 1). This suggests that PD patients have obvious overall cognitive decline, and peritoneal dialysis treatment may have an adverse impact on patients’ cognitive function, which is consistent with the findings of relevant studies indicating that chronic kidney disease and its related treatments can affect neurocognitive function.

**Table 1:** Neuropsychological data for the peritoneal dialysis patients and healthy controls included in the study.

Variables	HC (*n* = 49)	PD (*n* = 53)	*P*
SAS (score)	27.50 (26.25, 31.25)	38.75 (32.50, 41.25)	<0.01
SDS (score)	27.50 (25.00, 32.50)	42.50 (37.50, 51.25)	<0.01
PHQ-9 (score)	0.00 (0.00, 1.00)	5.00 (2.00, 8.00)	<0.01
MMSE (score)	29.00 (28.00, 30.00)	27.00 (22.00, 29.00)	<0.01
MoCA (score)	27.00 (25.00, 28.00)	24.00 (17.00, 27.00)	<0.01
Stroop-color (s)	39.17 (34.86, 46.07)	47.74 (37.57, 66.01)	<0.01
Stroop-word (s)	26.96 (22.19, 32.51)	33.16 (23.65, 43.67)	<0.01
Stroop-interference (s)	66.00 (58.75, 81.00)	80.13 (65.00, 106.89)	<0.01
SDMT (score)	39.49 ± 15.45	21.55 ± 15.54	<0.01
DST-forwards (score)	9.00 (9.00, 10.00)	6.00 (5.00, 8.00)	<0.01
DST-backwards (score)	6.00 (5.00, 7.00)	4.00 (3.00, 5.00)	<0.01
VFT (score)	12.00 (10.00, 15.00)	10.00 (8.00, 12.00)	<0.01
TMT-A (s)	62.00 (45.00, 84.00)	68.59 (49.10, 82.38)	0.55
TMT-B (s)	84.00 (67.00, 131.00)	83.82 (55.02, 100.48)	0.13

Values are presented as means ± SD or interquartile range or percentages.

PD, peritoneal dialysis; HC, healthy control; SAS, self-rating anxiety scale; SDS, self-rating depression scale; PHQ, patient health questionnaire; MMSE, mini-mental state examination; MoCA, montreal cognitive assessment; SDMT, symbol digit modalities test; DST, digit span test; TMT, trail making test; VFT, verbal fluency test.

### 3.2 Analysis of potential influencing factors for cognitive impairment

**Figure 1:**
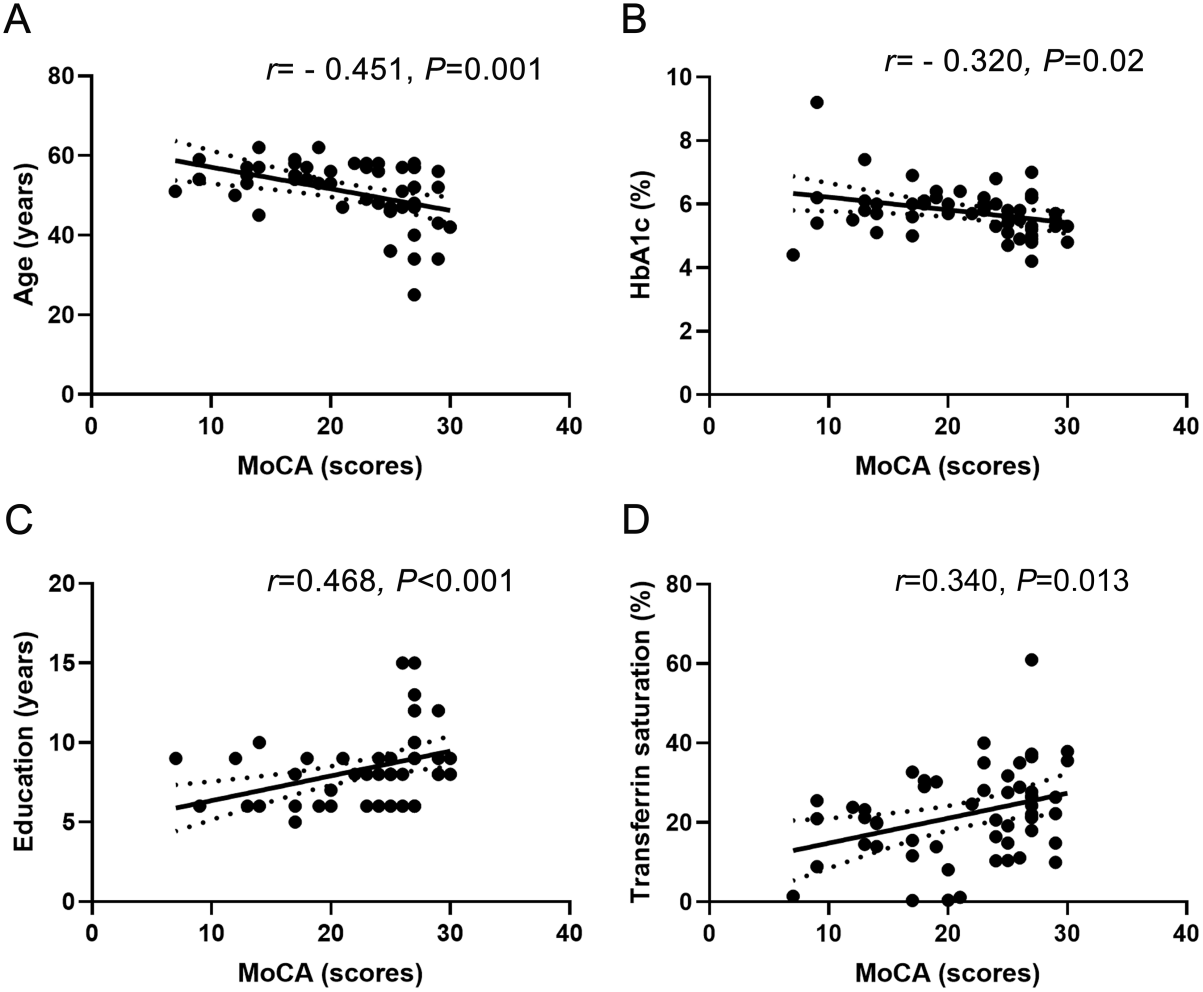
Correlation analysis between the clinical indicators and MoCA scores. **(A)** Age; **(B)** HbA1c; **(C)** Education; **(D)** Transferrin saturation. MoCA, montreal cognitive assessment; HbA1c, theglycosylated hemoglobin.

Compared with the NCI group, patients in the MCI group showed significantly higher levels of age, transferrin, and glycosylated hemoglobin. In contrast, their educational level and transferrin saturation were significantly lower (Table 2). No statistically significant differences were detected between the two subgroups in terms of other variables, including the primary etiologies of ESKD, use of various medications, TIV, and GMV (all *p* > 0.05). As shown in Figures 1A–D, MoCA score was negatively correlated with age (*r* = − 0.451, *p* = 0.001), and glycosylated hemoglobin level (*r* = − 0.320, *p* = 0.02). Conversely, it was positively correlated with educational level (*r* = 0.468, *p* < 0.001) and transferrin saturation (*r* = 0.340, *p* = 0.013).

**Table 2:** Demographical and clinical biochemical data for the peritoneal dialysis patients included in the study.

Variables	NCI (*n* = 24)	MCI (*n* = 29)	χ^2^/Z/t	*P*
Age (years)	46.04 ± 8.73	54.79 ± 4.18	−4.50	<0.001
Gender			0.85	0.356
Female [*n* (%)]	17 (70.83)	17 (58.62)	-	-
Male [*n* (%)]	7 (29.17)	12 (41.38)	-	-
Education (years)	9.00 (8.00, 10.50)	6.00 (6.00, 8.00)	−3.18	0.001
Dialysis period(months)	23.00 (11.00, 53.25)	27.00 (13.00, 69.00)	−0.93	0.353
Diabetes			0.11	0.743
No [*n* (%)]	20 (83.33)	22 (75.86)	-	-
Yes [*n* (%)]	4 (16.67)	7 (24.14)	-	-
Hypertension			0.52	0.472
No [*n* (%)]	3 (12.50)	1 (3.45)	-	-
Yes [*n* (%)]	21 (87.50)	28 (96.55)	-	-
Cause of ESKD [*n* (%)]			-	0.156
Chronic glomerulonephritis	11 (45.8)	12 (41.4)	-	-
IgA nephropathy	4 (16.7)	5 (17.2)	-	-
Hypertensive nephropathy	2 (8.3)	8 (27.6)	-	-
Diabetic kidney disease	2 (8.3)	3 (10.3)	-	-
Others and unknown	5 (20.8)	1 (3.4)	-	-
ACEI/ARBs [*n* (%)]	7 (29.2)	5 (17.2)	1.07	0.302
Alpha blockers [*n* (%)]	6 (25.0)	6 (20.7)	0.14	0.709
Beta blockers [*n* (%)]	6 (25.0)	9 (31.0)	0.24	0.627
Calcium channel blockers [*n* (%)]	18 (75.0)	25 (86.2)	-	0.482
Insulin use [*n* (%)]	3 (12.5)	6 (20.7)	-	0.487
Oral iron preparation [*n* (%)]	15 (62.5)	22 (75.9)	1.11	0.292
Erythropoietin use [*n* (%)]	20 (83.3)	22 (75.9)	-	0.725
Hemoglobin (g/L)	98.75 ± 15.26	102.59 ± 19.47	−0.79	0.436
Albumin (g/L)	37.17 ± 3.04	38.32 ± 3.53	−1.26	0.215
BUN (mmol/L)	21.59 (16.43, 23.23)	19.91 (17.71, 25.57)	−0.58	0.561
Uric acid (μmol/L)	431.92 ± 86.78	440.40 ± 78.07	−0.37	0.710
Serum creatinine (umol/L)	978.08 ± 243.15	964.67 ± 274.49	0.19	0.853
Calcium-phosphorus product	3.62 ± 1.00	4.06 ± 0.87	−1.71	0.093
HbA1c (%)	5.35 (4.97, 5.73)	6.00 (5.70, 6.20)	−3.06	0.002
CRP (mg/L)	1.14 (0.51, 8.66)	3.58 (1.34, 5.61)	−1.04	0.299
Transferrin(g/L)	1.77 ± 0.29	1.95 ± 0.34	−2.13	0.038
Transferrin saturation (%)	26.11 ± 11.34	18.70 ± 10.71	2.44	0.018
Total cholesterol (mmol/L)	4.65 (4.35, 4.90)	4.37 (3.76, 5.15)	−1.13	0.260
Triglyceride (mmol/L)	1.49 (1.16, 2.73)	1.68 (1.02, 3.54)	−0.04	0.971
LDL-C (mmol/L)	3.17 ± 0.63	2.88 ± 0.78	1.48	0.145
HDL-C (mmol/L)	1.16 ± 0.28	1.17 ± 0.31	−0.15	0.880
VLDL-C (mmol/L)	0.55 (0.43, 1.01)	0.62 (0.38, 1.31)	−0.06	0.950
iPTH (pg/ml)	192.00 (95.25, 391.25)	259.00 (165.00, 391.00)	−0.79	0.430
24-h residual urine volume (L)	0.1 (0.0, 0.4)	0.0 (0.0, 0.4)	−0.78	0.436
Total Kt/V	1.99 (1.87, 2.26)	1.91 (1.31, 2.07)	−1.71	0.088
Total CCr (L/Week)	51.84 (41.34, 71.98)	50.58 (45.61, 53.48)	−0.13	0.900
TIV (cm3)	1431.13 ± 136.89	1435.55 ± 152.96	−0.11	0.913
GMV (cm3)	585.21 ± 50.79	599.72 ± 61.42	−0.93	0.359

Values are presented as means ± SD or interquartile range or percentages.

MCI, mild cognitive impairment; NCI, non-cognitive impairment; ESKD, end-stage kidney disease; ACEI, angiotensin converting enzyme inhibitor; ARBs, angiotensin-receptor blockers; BUN, blood urea nitrogen; HbA1c, glycosylated hemoglobin; CRP, c-reactive protein; LDL-C, low-density lipoprotein cholesterol; HDL-C, high-density lipoprotein cholesterol; VLDL-C, very low-density lipoprotein cholesterol; iPTH, intact parathyroid hormone; CCr, creatinine clearance rete; Kt/V, urea clearance index; TIV, total intracranial volume; GMV, gray matter volume.

### 3.3 Results of brain entropy and functional connectivity

**Figure 2:**
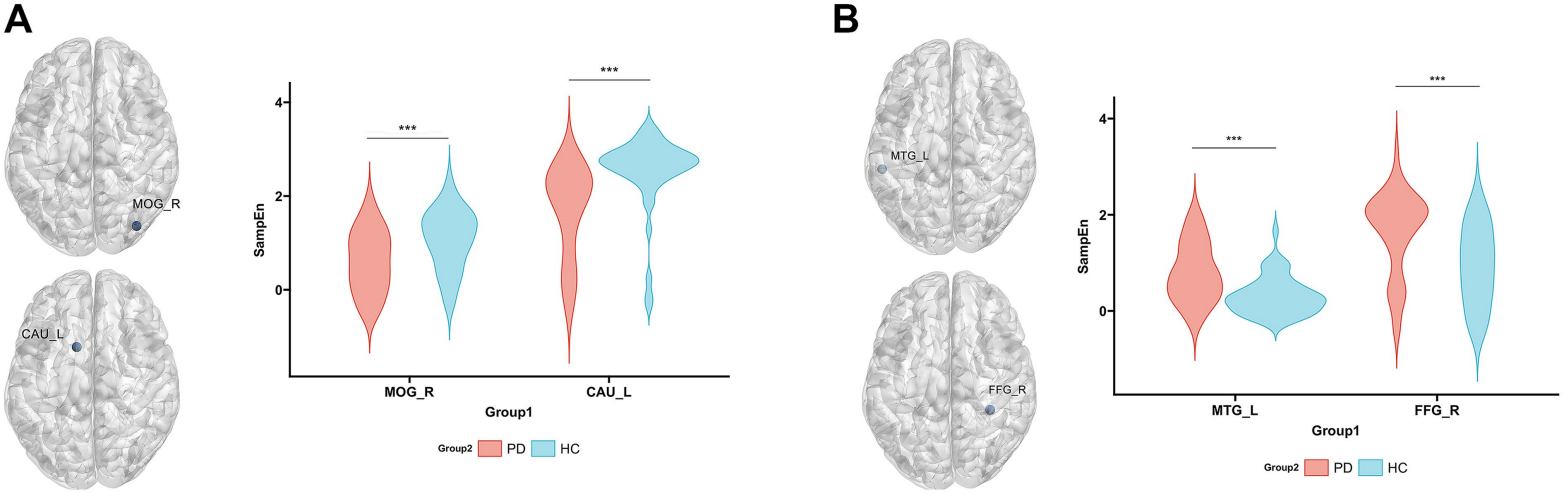
Differences in brain entropy values among PD and HC groups. **(A)** PD group exhibited significantly decreased brain entropy values in the right MOG and left CAU; **(B)** PD group exhibited significantly increased brain entropy values in the left MTG and right FFG. PD, peritoneal dialysis; HC, healthy control; MOG, middle occipital gyrus; CAU, caudate nucleus; MTG, middle temporal gyrus; FFG, fusiform gyrus.

In comparison with the normal control group, the PD group exhibited significantly decreased brain entropy values in the right middle occipital gyrus and left caudate nucleus (Figure 2A), while increased values were observed in the left middle temporal gyrus and right fusiform gyrus (Figure 2B). These four brain regions were designated as ROI 1 to ROI 4, respectively, for whole - brain resting - state functional connectivity analysis. Compared with healthy subjects, there were no statistically significant differences in functional connectivity between ROI 2 to ROI 4 and the whole brain in the PD group. When the right middle occipital gyrus, a brain region with differential entropy values, was set as ROI 1 for calculating functional connectivity with the whole brain and a two sample t - test was conducted, it was found that in PD patients, the functional connectivity between the right middle occipital gyrus and three brain regions, namely the right fusiform gyrus, right middle frontal gyrus, and right precuneus, was reduced (Table 3).

**Table 3:** The result of whole brain RSFC analysis with rMOG as ROI between PD and HC groups.

Brain regions	Peak MNI coordinate			Cluster size	T
	X	Y	Z		
FFG_R	−15	−30	−3	131	−6.3493
MFG_R	27	12	60	176	−4.6587
PCUN_R	3	−45	72	226	−4.9915

RSFC, resting-state functional connectivity; ROI, region of interest; PD, peritoneal dialysis; HC, healthy control; MIN, Montreal Neurological Institute; MOG, middle occipital gyrus; FFG, fusiform gyrus; MFG, middle frontal gyrus; PCUN, precuneus.

### 3.4 Correlation analysis

In the PD group, the brain entropy value of the right middle occipital gyrus was significantly negatively correlated with the scores of the SAS (*r* = − 0.304, *p* = 0.027) and the SDS (*r* = − 0.418, *p* = 0.002). The brain entropy value of the left middle temporal gyrus was positively correlated with the scores of the SAS (*r* = 0.430, *p* = 0.001), SDS (*r* = 0.352, *p* = 0.010), and PHQ - 9 (*r* = 0.491, *p* < 0.001), and it was also correlated with the abstract dimension score of the MoCA scale (*r* = 0.286, *p* = 0.038). Resting - state functional connectivity analysis revealed that the functional connectivity value between the right fusiform gyrus and the right middle occipital gyrus was significantly positively correlated with the orientation (*r* = 0.290, *p* = 0.035) and construction ability (*r* = 0.274, *p* = 0.047) scores of the MMSE.

**Figure 3:**
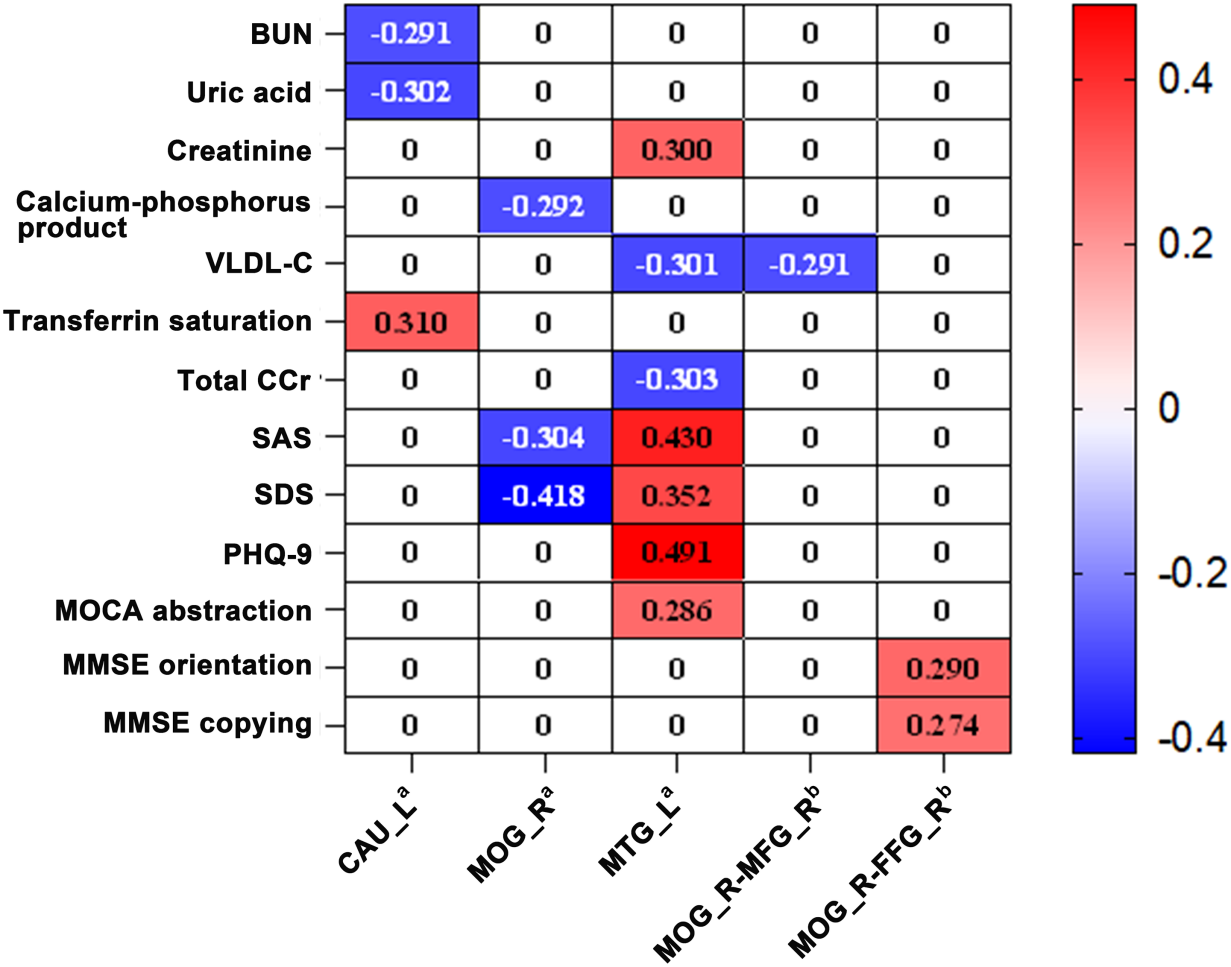
Heatmap of correlation analysis between brain biomarkers and clinical data. BUN, blood urea nitrogen; VLDL-C, very low-density lipoprotein cholesterol; CCr, creatinine clearance rete; SAS, self-rating anxiety scale; SDS, self-rating depression scale; MoCA, montreal cognitive assessment; MMSE, mini-mental state examination; MOG, middle occipital gyrus; CAU, caudate nucleus; MTG, middle temporal gyrus; MFG, middle frontal gyrus; FFG, fusiform gyrus. a: brain entropy values in differential brain regions. b: Functional connectivity with the right middle occipital gyrus; 0: no correlation between variables.

The associations between the brain entropy values of differential brain regions, the functional connectivity values of the right middle occipital gyrus, and clinical indicators also have clinical significance. The brain entropy value of the left caudate nucleus was positively correlated with transferrin saturation (*r* = 0.310, *p* = 0.024) and negatively correlated with urea nitrogen (*r* = − 0.291, *p* = 0.035) and uric acid (*r* = − 0.302, *p* = 0.028). The brain entropy value of the right middle occipital gyrus was negatively correlated with the calcium - phosphorus product (*r* = − 0.292, *p* = 0.034). The brain entropy value of the left middle temporal gyrus was positively correlated with creatinine (*r* = 0.300, *p* = 0.029) and negatively correlated with very low - density lipoprotein (*r* = − 0.301, *p* = 0.029) and total CCr (*r* = − 0.303, *p* = 0.027). Additionally, the functional connectivity value between the right middle frontal gyrus and the right middle occipital gyrus was negatively correlated with very low - density lipoprotein (*r* = − 0.291, *p* = 0.034). These results are presented in Figure 3.

**Figure 4:**
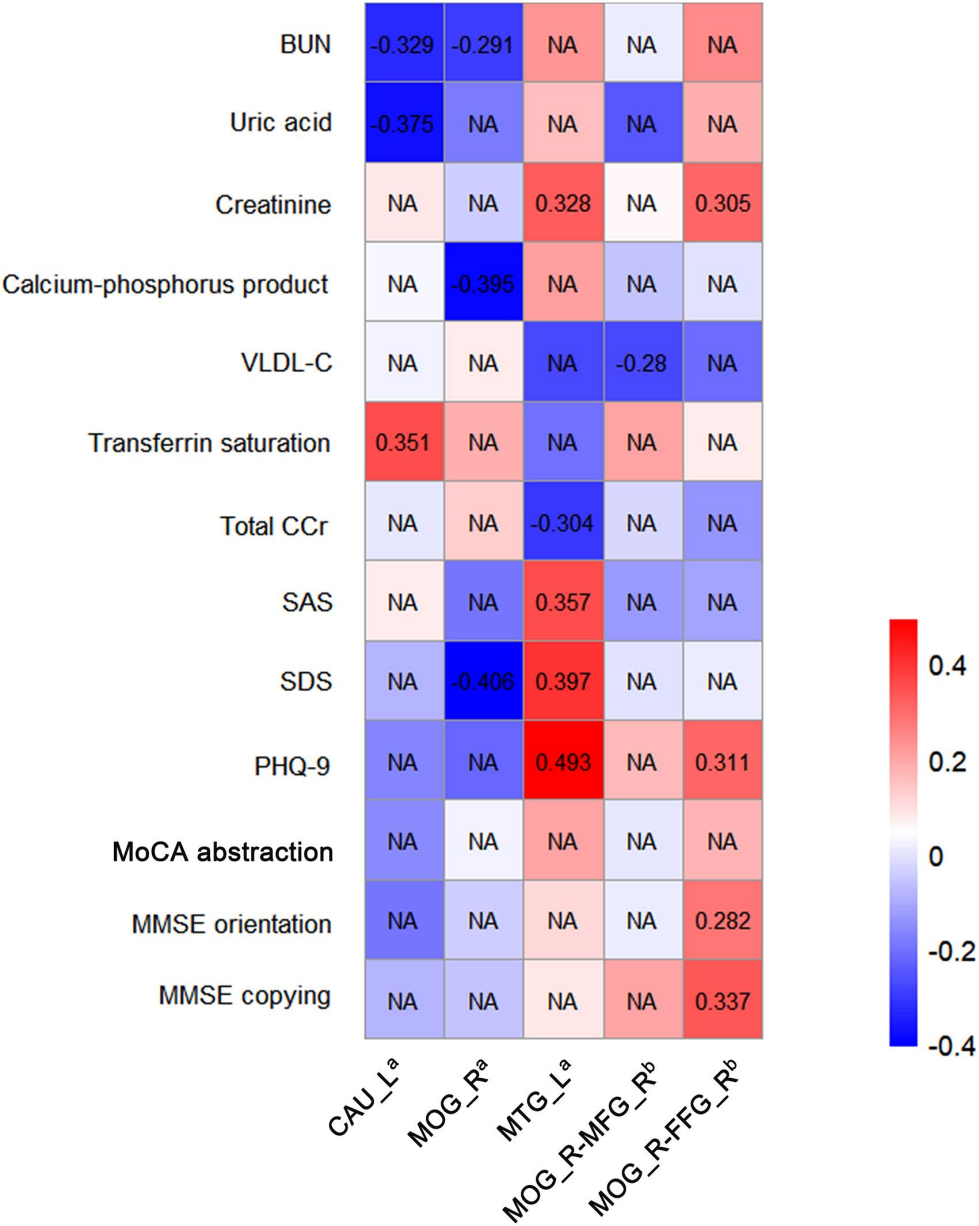
Partial correlation heatmap of brain entropy and functional connectivity with clinical parameters (adjusted for age, educational level and gray matter volume). BUN, blood urea nitrogen; VLDL-C, very low-density lipoprotein cholesterol; CCr, creatinine clearance rete; SAS, self-rating anxiety scale; SDS, self-rating depression scale; MoCA, montreal cognitive assessment; MMSE, mini-mental state examination; MOG, middle occipital gyrus; CAU, caudate nucleus; MTG, middle temporal gyrus; MFG, middle frontal gyrus; FFG, fusiform gyrus. a: brain entropy values in differential brain regions. b: Functional connectivity with the right middle occipital gyrus.

Partial correlation analyses controlling for age, education, and gray matter volume confirmed that the main correlations between brain entropy, functional connectivity and clinical metabolic, emotional and cognitive indicators remained statistically significant. Detailed coefficients are presented in Figure 4.

## 4 Discussion

In this study, we employed rs - fMRI to conduct an in - depth investigation into the abnormal changes in brain entropy and functional connectivity in PD patients. The results revealed that PD patients exhibited significantly decreased brain entropy values in the right middle occipital gyrus and left caudate nucleus, while increased entropy values were observed in the left middle temporal gyrus and right fusiform gyrus. Additionally, the functional connectivity between the right middle occipital gyrus and the right fusiform gyrus, right middle frontal gyrus, and right precuneus was weakened in PD patients. Through the analysis of neuropsychological scales, it was clearly determined that PD patients suffer from multi - dimensional cognitive impairment. By combining general demographic data and laboratory indicators, potential influencing factors were explored. Among them, age, educational level, glycosylated hemoglobin, and transferrin saturation were found to be correlated with cognitive impairment in PD patients. Meanwhile, in the PD group, the brain entropy value of the right middle occipital gyrus was negatively correlated with the scores of the SDS. The entropy value of the left middle temporal gyrus was positively correlated with the scores of the SAS, SDS, and PHQ - 9. Furthermore, the functional connectivity value between the right fusiform gyrus and the right middle occipital gyrus was significantly positively correlated with the scores of the orientation and structural ability dimensions of the MMSE scale. Importantly, the correlations between brain entropy/RSFC values and metabolic parameters (e.g., BUN, creatinine, calcium-phosphorus product, HbA1c) indicate that the observed brain functional changes may be largely driven by the systemic illness—ESKD with its uremic, inflammatory, metabolic disturbances and long-term peritoneal dialysis.

PD patients scored significantly lower than the healthy control group in multiple cognitive tests such as MoCA and MMSE, showing multi - dimensional cognitive impairments in memory, attention, executive function, and other aspects. Due to its high sensitivity and specificity, the MoCA score was used in this study to evaluate the overall cognitive function of PD patients (Salazar-Felix et al., 2021). The prevalence of CI was 54.7%, which is consistent with previous studies (Aiumtrakul et al., 2024; Shi et al., 2024). At the same time, correlation analysis showed that age, educational level, glycosylated hemoglobin, and other factors were related to cognitive impairment in PD patients. It is known that the risk factors for cognitive impairment in PD patients include advanced age (Wang et al., 2022), diabetes mellitus (Zhu et al., 2023), low educational level (Shea et al., 2016; Wang et al., 2022; Zhu et al., 2023), elevated levels of high sensitivity C - reactive protein (hs - CRP), etc. Some studies have shown that iPTH is related to cognitive function (Kalaitzidis et al., 2013), and there is a U - shaped relationship between them. Both excessively high and low levels of iPTH significantly affect cognitive function (Jiang et al., 2020; Mobushar et al., 2025). In addition, the prevalence of hypertension in PD patients in this study reached 92.45%. Although no statistical association was found between hypertension and CI, it may increase the risk of cerebrovascular diseases and accelerate the progression of cognitive impairment.

The pathogenesis of cognitive impairment in patients with ESKD receiving peritoneal dialysis is multifactorial and may be related to the effects of uremic toxins, inflammatory status, impaired neuronal function, cerebral blood flow autoregulation disorders, calcium - phosphorus metabolism disorders, and genetic factors (Drew et al., 2019; Viggiano et al., 2020). At present, many studies have conducted in - depth discussions on possible pathogenesis and influencing factors, attempting to analyze the complex pathophysiological processes behind it, in order to provide a theoretical basis for prevention and treatment.

Brain entropy (BEN) is a non - linear index representing the complexity of brain activity and also indicates the complexity of information processing in the brain. In this study, compared with normal controls, PD patients showed significantly decreased brain entropy values in the right middle occipital gyrus and left caudate nucleus, and increased values in the left middle temporal gyrus and right fusiform gyrus. These changes in brain entropy values in these brain regions indicate that the resting - state brain of PD patients has undergone changes in functional temporal dynamics, providing new information for certain functional changes in PD patients. Functional connectivity reflects the correlation of neuronal activities between different brain regions by analyzing the time - series data of activation of different neurons in the brain. Compared with the healthy control group, PD patients had weakened functional connectivity between the right middle occipital gyrus and the right fusiform gyrus, right middle frontal gyrus, and right precuneus. The right middle occipital gyrus is a key node for visual information integration, while the fusiform gyrus is responsible for the in - depth processing of visual signals to maintain basic visual cognitive functions (such as spatial structure recognition, object orientation, etc.) (Pourtois et al., 2009; Zhang et al., 2018). Correlation analysis showed that the functional connectivity value between the right fusiform gyrus and the right middle occipital gyrus was significantly positively correlated with the scores of the orientation and structural ability dimensions of the MMSE scale, suggesting that the weakened connectivity may lead to impairment in the corresponding cognitive dimensions. Cognitive function impairment in patients with ESKD mainly manifests in executive ability, attention, information processing ability, memory, motor function, etc., especially the most serious impairment in executive ability and memory (Huang et al., 2021; Sanchez-Fernandez et al., 2018). It should be noted that the precuneus is included in the default mode network (DMN), which is a brain region activated in correlation with attention and cognition when the brain is in a resting state (Raichle, 2015). The middle frontal gyrus is a key component of the Central Executive Network (CEN), which is responsible for goal - directed cognitive tasks. SU et al. reported that in maintenance hemodialysis patients, the brain regions with abnormal spontaneous brain activity and functional integration disorders are mainly located in the DMN region (Su et al., 2021). CAO et al. found that the FC between DMN, salience network, and central executive network was weakened in hemodialysis patients (Cao et al., 2021). Combined with the phenomenon of weakened connectivity between the right middle occipital gyrus and the central executive network (middle frontal gyrus) and default mode network (precuneus) in this study, it suggests that PD patients not only have functional reorganization within the visual - related cortex but also may be accompanied by abnormal cross - network functional integration. However, the specific impact on cognitive function still needs further exploration.

Studies have shown that depression, including clinical depression and subclinical depression, is likely to lead to cognitive impairment. Among hemodialysis patients, those with more severe depressive symptoms showed significantly poorer performance in tests assessing processing speed, attention, and executive function (Agganis et al., 2010). This study further found that there is a region-specific association between emotional abnormalities and changes in brain function in patients with PD. In addition to the correlation between reduced brain entropy in the right middle occipital gyrus and negative emotions, the brain entropy value in the left middle temporal gyrus was also associated with abstract dimensions of various emotional scales and cognitive scales. This suggests that abnormal functional complexity of the temporal lobe may be the common neural basis for emotional disorders and decreased abstract cognitive ability. Notably, a study on generalized anxiety disorder showed that patients had increased brain entropy levels in the right middle occipital gyrus (Fan et al., 2023). However, in this study, PD patients exhibited decreased brain entropy in this brain region, which was associated with emotions. This difference may be related to the unique metabolic microenvironment of PD, such as calcium-phosphate disorders and accumulation of oxidative stress.

Several limitations of the present study should be acknowledged. First, this is a single-center cross-sectional study, which cannot establish causal relationships between altered brain function and clinical characteristics. The observed brain entropy and functional connectivity changes, as well as cognitive impairment, are joint outcomes of multiple factors including ESKD, uremic toxins, chronic metabolic disorders and peritoneal dialysis. Therefore, we cannot attribute these abnormalities solely to peritoneal dialysis. Second, although we supplemented data on the primary etiologies of ESKD and regular medications, and adjusted cognition-related indicators as covariates during statistical analyses to control confounding effects, the combined impacts of various medications and systemic metabolic disturbances could not be completely ruled out. This is a potential source of bias in our study. Third, we strictly excluded patients with cerebrovascular diseases, clinically diagnosed mental disorders and suspected neurodegenerative diseases during enrollment based on medical records and brain MRI. However, the possibility of mild or subclinical cerebral vascular lesions and early neurodegenerative changes in a small number of participants cannot be fully excluded. Subclinical emotional symptoms were assessed using standardized scales and incorporated into correlation analyses, yet their long-term influence remains to be further explored. Fourth, we did not perform detailed subgroup imaging comparisons between patients with and without mild cognitive impairment, as subgroup division would lead to reduced sample size and unstable statistical results. In addition, the mean age of our participants was approximately 50 years, which is younger than the average age of ESKD patients reported in most studies. This is associated with regional dialysis selection habits and our strict inclusion and exclusion criteria. Therefore, the present results cannot be fully generalized to elderly PD patients over 70 years old. Finally, we did not perform vertex-wise cortical thickness analyses; future surface-based morphometry studies are warranted.

## 5 Conclusion

In conclusion, this study applied resting-state functional magnetic resonance imaging to investigate changes in brain entropy in PD patients and further analyzed the corresponding changes in functional connectivity in brain regions with altered brain entropy. The abnormal brain function in PD patients is a combined result of ESKD itself, chronic metabolic disturbance, uremic toxins and long-term peritoneal dialysis, providing new insights into the pathogenesis of cognitive impairment in PD patients and the exploration of potential intervention targets.

## Data Availability

The original contributions presented in the study are included in the article/supplementary material, further inquiries can be directed to the corresponding author/s.
